# Lung cancer mortality clusters in Shandong Province, China: how do they change over 40 years?

**DOI:** 10.18632/oncotarget.21144

**Published:** 2017-09-21

**Authors:** Zhentao Fu, Yingmei Li, Zilong Lu, Jie Chu, Jiandong Sun, Jiyu Zhang, Gaohui Zhang, Fuzhong Xue, Xiaolei Guo, Aiqiang Xu

**Affiliations:** ^1^ Department for Chronic and Non-Communicable Disease Control and Prevention, Shandong Center for Disease Control and Prevention, Jinan, China; ^2^ The Second People's Hospital of Jinan, Jinan, China; ^3^ School of Public Health and Social Work, Queensland University of Technology, Brisbane, Australia; ^4^ School of Public Health, Shandong University, Jinan, China

**Keywords:** lung cancer, spatial scan statistics, mortality, epidemiology

## Abstract

Lung cancer has long been a major health problem in China. This study aimed to examine the temporal trend and spatial pattern of lung cancer mortality in Shandong Province from 1970 to 2013. Lung cancer mortality data were obtained from Shandong Death Registration System and three nationwide retrospective cause-of-death surveys. A Purely Spatial Scan Statistics method with Discrete Poisson models was used to detect possible high-risk spatial clusters. The results show that lung cancer mortality rate in Shandong Province increased markedly from 1970-1974 (7.22 per 100,000 person-years) to 2011-2013 (56.37/100, 000). This increase was associated with both demographic and non-demographic factors. Several significant spatial clusters with high lung cancer mortality were identified. The most likely cluster was located in the northern region of Shandong Province during both 1970-1974 and 2011-2013. It appears the spatial pattern remained largely consistent over the last 40 years despite the absolute increase in the mortality rates. These findings will help develop intervention strategies to reduce lung cancer mortality in this large Chinese population.

## INTRODUCTION

Lung cancer is the most frequently diagnosed cancer and the most common cause of cancer-related death worldwide [1, 2]. It is estimated that there were 1.8 million new cases in 2012, 58% of which occurred in less developed regions. In 2012, lung cancer took the lives of about 1.59 million people worldwide, almost one in every five cancer caused deaths (19.4%) in the world [2]. There is a large variation in lung cancer incidence and mortality rate in both men and women across the world [3]. In most western countries, the lung cancer incidence and mortality rate have been decreasing. For example, in the United States, the lung cancer incidence decreased significantly among men during 2004-2009, although it remained stable among women during the same period [4]. In contrast, the lung cancer incidence and mortality rates still increase rapidly in China during the past several decades. In addition to the high incidence and mortality rate, lung cancer also presents high lethality. The 5-year survival rate for lung cancer patients is lower than those for other types of cancer, such as colon, breast, and prostate cancers [5, 6].

Shandong Province, which has a population of more than 96 million (nearly twice the population of South Korea) is the second most populated province in China. Shandong is also one of the provinces with the fastest growth rate in lung cancer mortality in China [7, 8]. The rank of lung cancer in cancer mortality among the types of cancer that cause death went up from the 5^th^ in the 1970s to the 1^st^ during 2004-2005 [9]. Lung cancer is sometimes found to be concentrated in some specific regions [10]. Identifying these regions will help to identify the pathogenic factors of lung cancer and targeted strategies for preventing and controlling this highly fatal disease.

Previous studies have suggested that there were variations in spatial distribution of lung cancer in different regions in China. These studies are usually based on a samples from surveillance sites or retrospective sampling survey of causes of death in some specific regions (different separated counties) of China [11–13]. Data in a few separated counties was used to represent the whole province in these studies. However, no studies in China have explored the clusters of lung cancer mortality based on surveillance data from an entire province, especially a province that has a large population (e.g., nearly 100 million, or 8 percent of China’s population). In addition, to the best of our knowledge, no studies have examined the change of clustering regions of lung cancer mortality at county level over different eras (e.g., over 40 years) using Spatial Scan Statistics. Furthermore, there is limited published information describing the disease burden and spatial distribution of lung cancer in Shandong, China. Such information may be important for planning cancer control and prevention activities.

To remedy this deficiency, this study was designed to determine the spatial distribution and high risk areas of lung cancer mortality based on the whole population at the county level in Shandong, China, and examine the difference in spatial distribution of lung cancer mortality between the time period of 2011-2013 and the time period of 1970-1974.

## RESULTS

### Mortality trend of lung cancer in Shandong Province from 1970 to 2013

**Figure 1:**
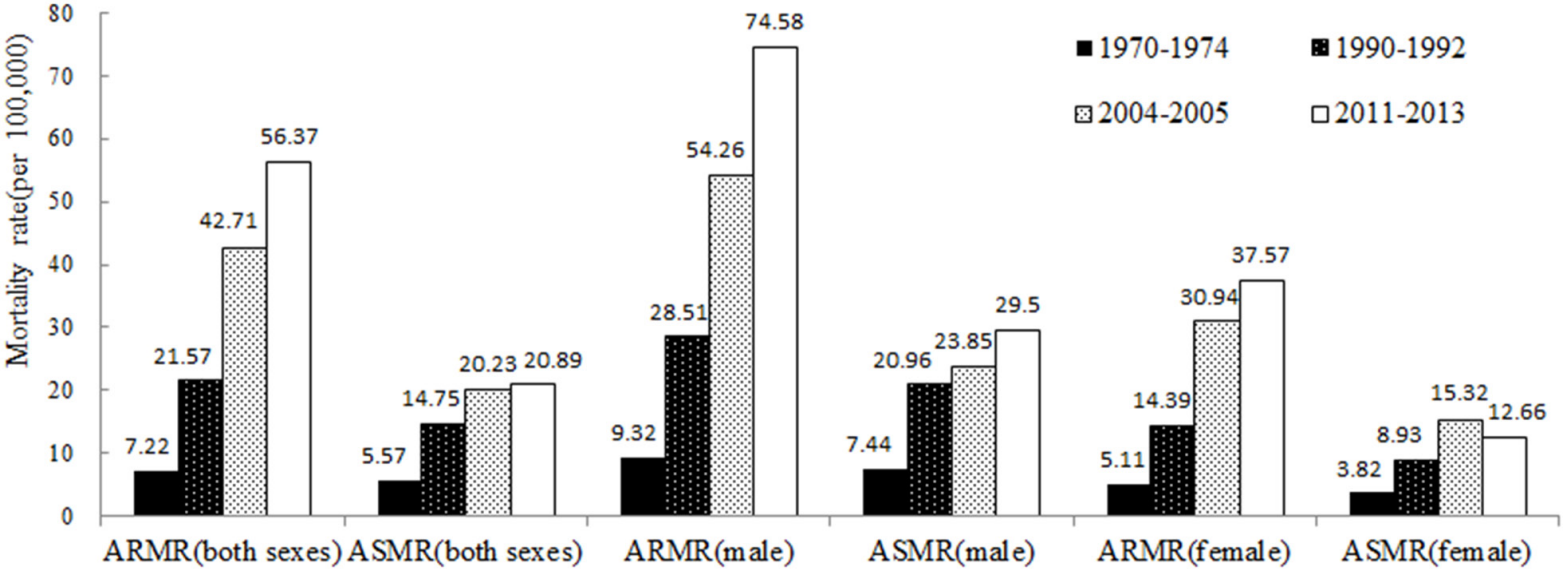
ARMR and ASMR of lung cancer in Shandong Province for different eras

The analysis results show a significant increasing trend in lung cancer mortality rate in Shandong Province from 1970 to 2013. During 1970-1974, the lung cancer crude mortality rate in Shandong Province was 7.22/100,000. It increased to 56.37/100,000 during 2011-2013, nearly 7.81 times of that during 1970-1974. Lung cancer mortality during 2011-2013 was 161.34% and 31.98% higher than that during 1990-1992(21.57/100,000) and 2004-2005(42.71/100,000), respectively. During 1970-1974, the age adjusted lung cancer mortality rate standardized according to the age structures of Chinese population in 1964 (ASMR) was 5.57 per 100,000. During 1990-1992, 2004-2005 and 2011-2013, the ASMRs were 14.75/100,000, 20.23/100,000 and 20.89/100,000, respectively. Thus, with age adjustment, the lung cancer mortality still increased from 1970 to 2013 as shown in Figure 1. Although the average reported mortality rate (ARMR) and ASMR of lung cancer in male were much higher than those in female, the increasing trends were basically the same for male and female. The only difference was that the ARMR of lung cancer in female had increased from 2004-2005 to 2011-2013, while the ASMR of lung cancer in female had dropped during the same period.

**Figure 2:**
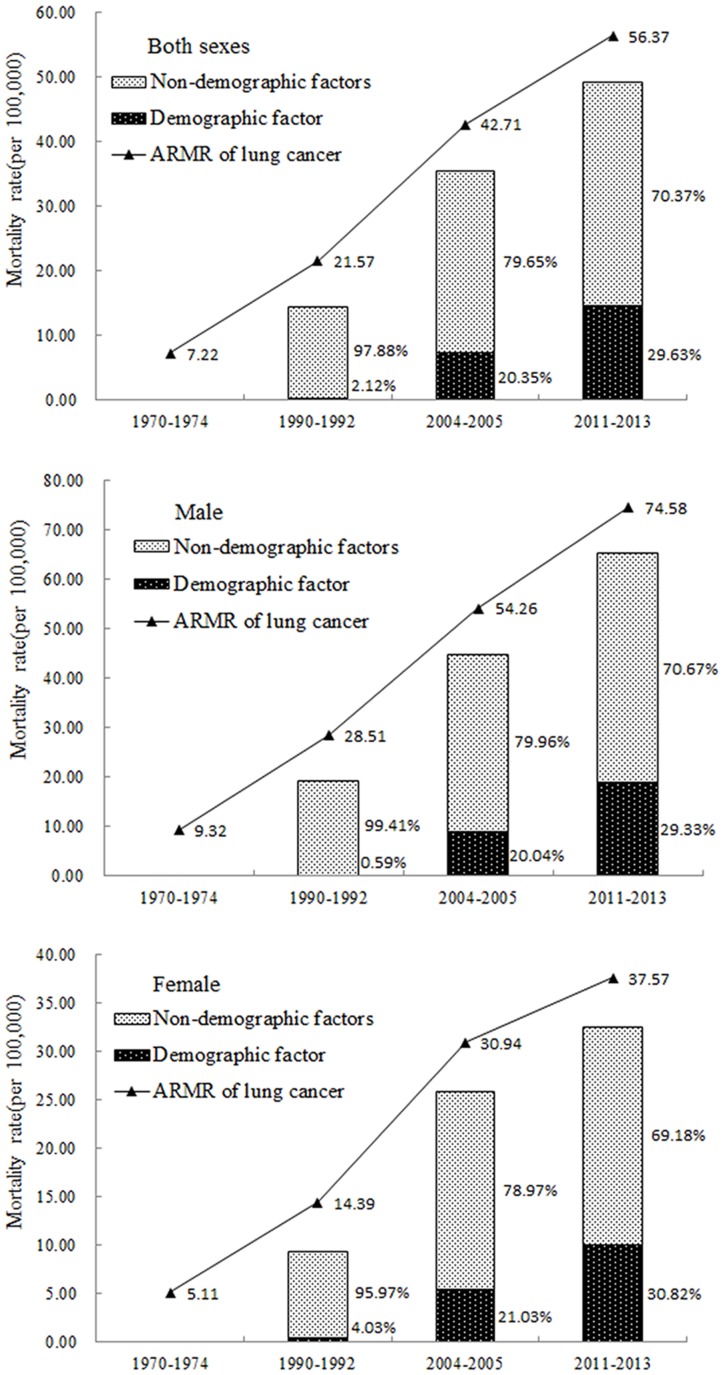
Decomposition analysis of the difference in lung cancer mortality rate for different eras in Shandong Province

Using a decomposition analysis of the difference in lung cancer mortality of different eras, we found out that the mortality rate of lung cancer was increasing over the years in Shandong, and the main reason for the increase was non-demographic factors (including smoking, air pollution and so on). The non-demographic factors contributed to about 97.88%, 79.65% and 70.37% of the increase in lung cancer mortality rate in 1990-1992, 2004-2005 and 2011-2013, respectively. Demographic factor (population aging) contributed to about 2.12%, 20.35% and 29.63% of the increase in 1990-1992, 2004-2005 and 2011-2013, respectively. Although the proportion of contribution caused by the demographic factors had increased over time, the non-demographic factors were still the dominant factors for the increase in lung cancer mortality (Figure 2). Same conclusion could be reached from the results for both male and female.

### Spatial distribution of lung cancer mortality rate in Shandong Province

**Figure 3:**
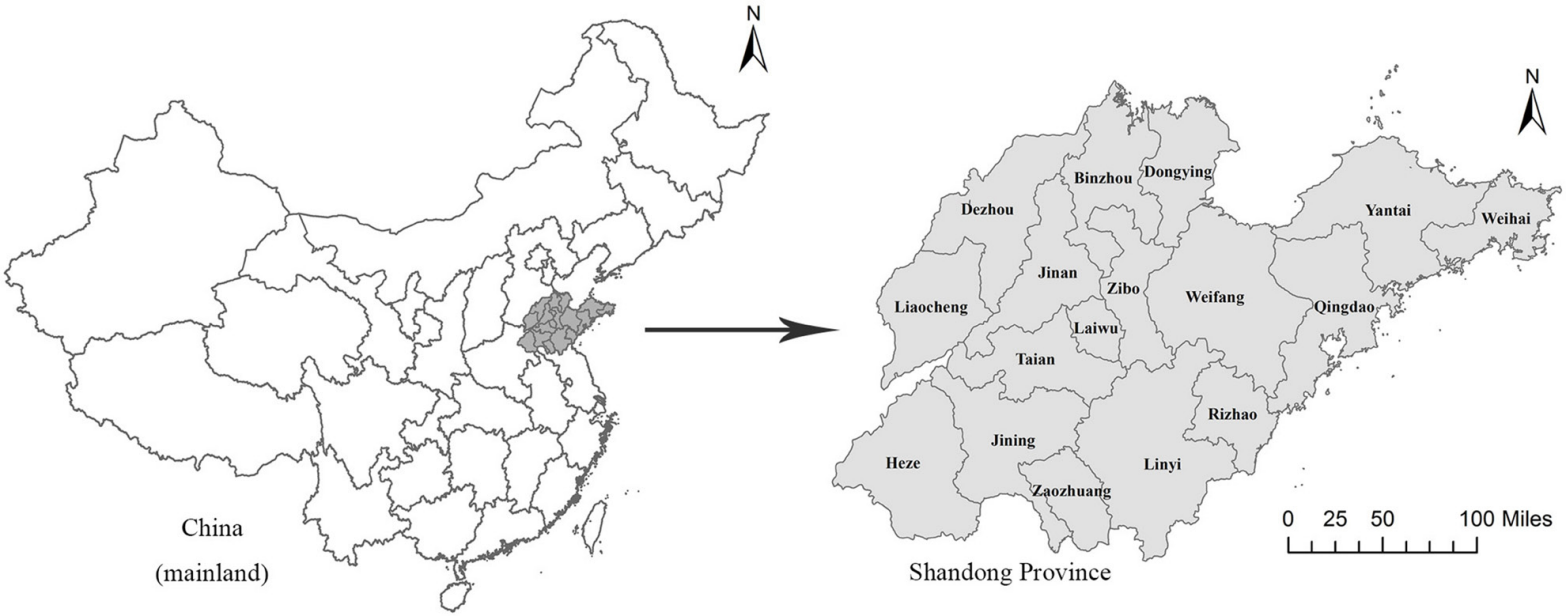
Location of Shandong Province, China ArcGis 10.2 (http://www.esri.com/software/arcgis/arcgisonline).

**Figure 4:**
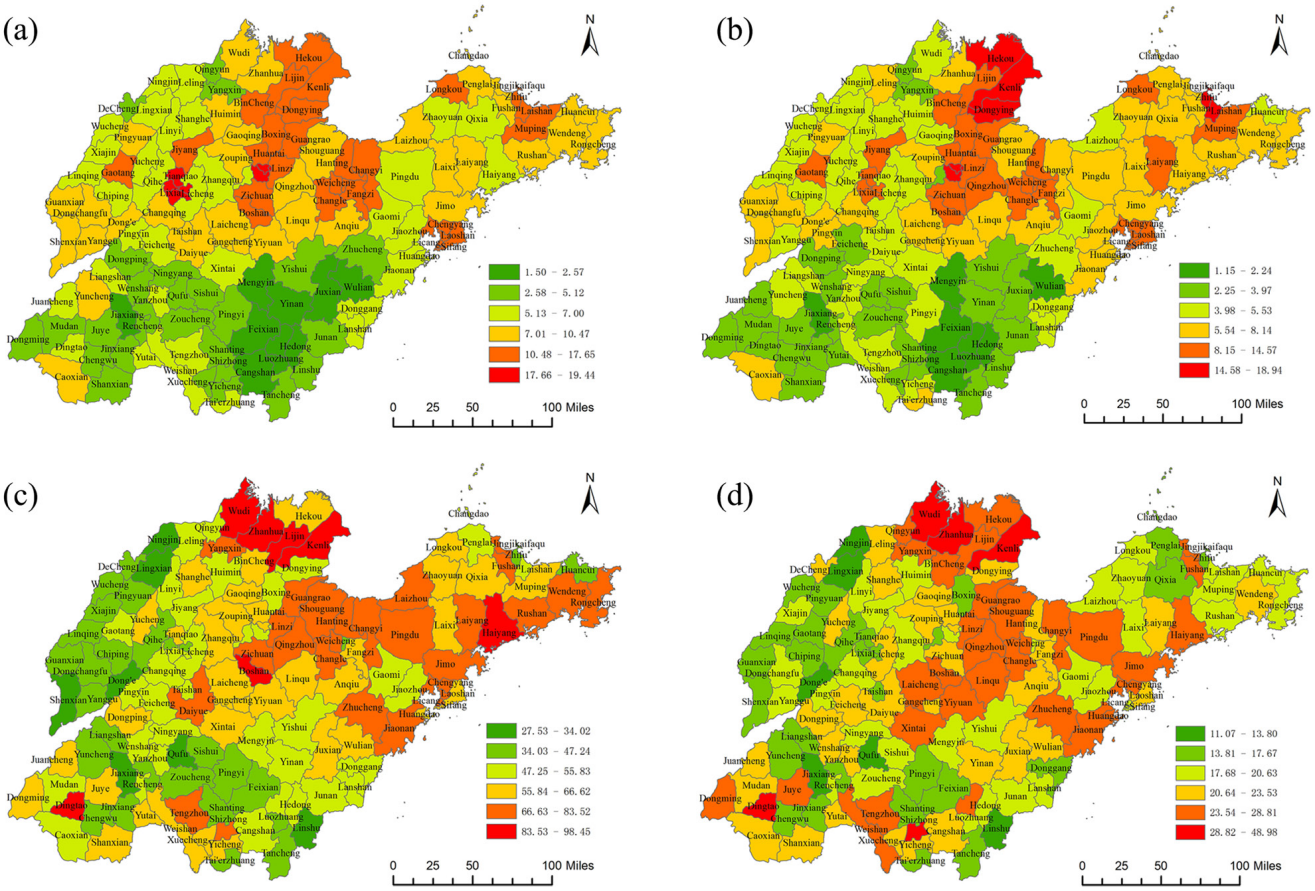
Spatial distribution of lung cancer mortality at the county level in Shandong Province during 1970-1974 and 2011-2013 **(a)** 1970-1974(ARMR), **(b)** 1970-1974(ASMR), **(c)** 2011-2013(ARMR), **(d)** 2011-2013(ASMR) ArcGis 10.2 (http://www.esri.com/software/arcgis/arcgisonline).

Figure 3 shows the geographic location of Shandong Province in China, which contains 17 prefecture-level cities. Figure 4 shows the spatial distribution of lung cancer mortality rate in Shandong Province. In Figure 4, mortality rates lower than the expected mortality rate are represented by the green color, while mortality rates higher than the expected mortality rate are represented by the red color. In 1970-1974, the lung cancer mortality rate in the northern areas was higher than that in the southern areas of Shandong Province (Figure 4a). After age adjustment, the spatial distribution of lung cancer mortality rate had little change (Figure 4b). The high mortality rates mainly occurred in the northern region of Shandong, including some counties in the cities of Dongying, Weifang, Zibo, Jinan, Yantai and Qingdao.

However, during 2011-2013, the mortality rates of lung cancer were all over 20 per 100,000 in all counties in Shandong (Figure 4c). The counties having high mortality rates were mainly concentrated in the northern and eastern regions and several counties of the southwestern region in Shandong. Some counties in the northern region of Shandong remained as areas with high lung cancer mortality rate. A few counties in the southwestern region that previously had a low lung cancer mortality rate became hotspots in Shandong. After age adjustment (Figure 4d), the mortality rates of lung cancer were no longer high in some counties of the eastern region, such as Laizhou County, Rushan County and Rongcheng County.

### Spatial scan statistical analysis

**Figure 5:**
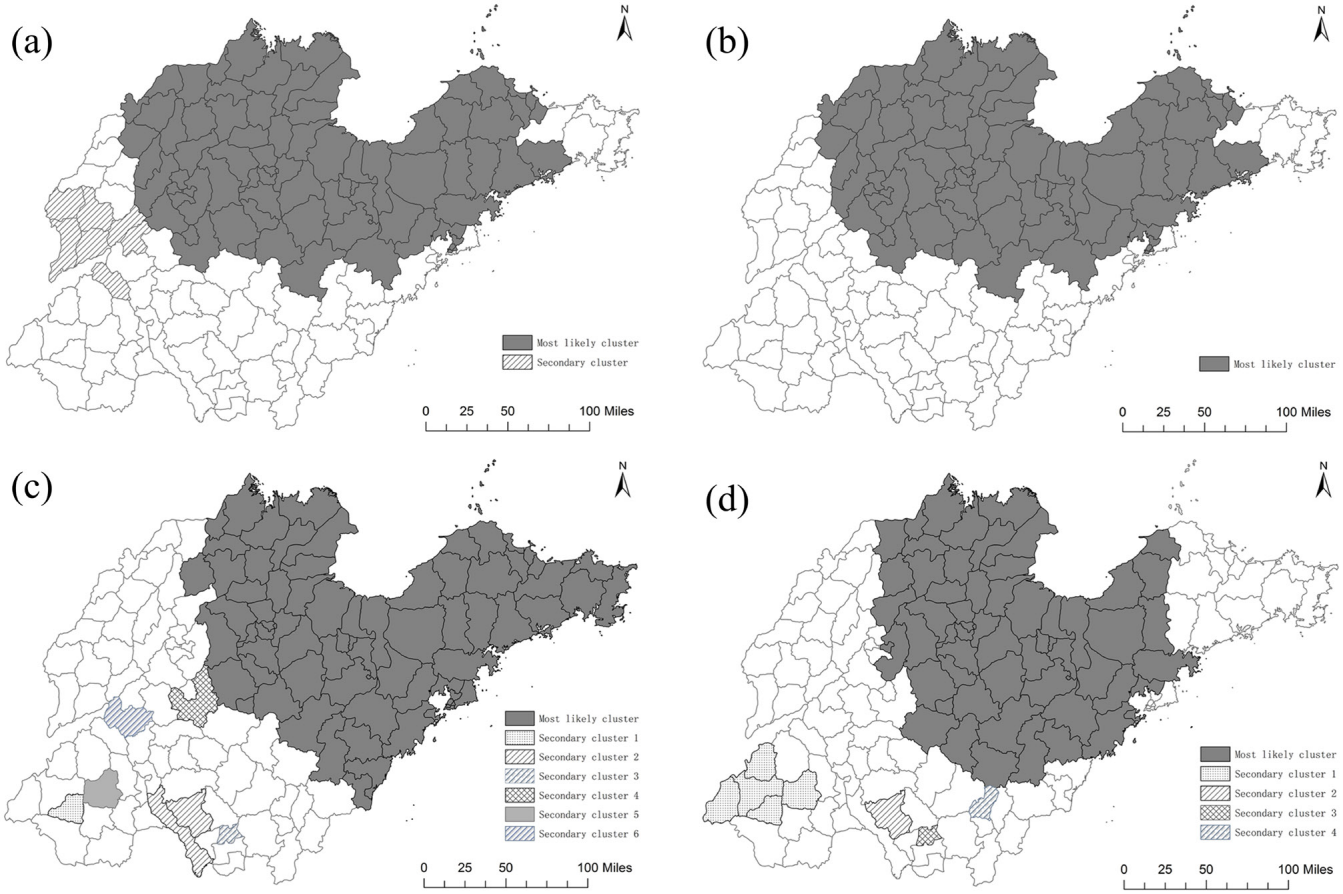
Lung cancer mortality clusters at the county level in Shandong during 1970-1974 and 2011-2013 using spatial scan statistical analysis **(a)** 1970-1974(ARMR), **(b)** 1970-1974(ASMR), **(c)** 2011-2013(ARMR), **(d)** 2011-2013(ASMR) ArcGis 10.2 (http://www.esri.com/software/arcgis/arcgisonline).

The spatial scan statistical analysis revealed two significant spatial clustering areas of lung cancer mortality in the whole province during the period from 1970 to 1974 (Table 1 and Figure 5a), suggesting that lung cancer mortality was not randomly distributed. The most likely cluster was located in the northern region of Shandong Province around Bohai Bay, including 77 counties with a relative risk (RR) of 1.81 compared to the rest of Shandong Province. The secondary cluster was located in the western region (including 7 counties) with a RR of 1.15 compared to the other areas in Shandong Province. After age adjustment, the most likely cluster did not change, still including the same 77 counties and with a relative risk (RR) of 1.96 compared to the rest of Shandong Province. The secondary cluster disappeared after age adjustment (Table 1 and Figure 5b).

**Table 1:** Results of the spatial scan analysis of lung cancer mortality at the county level in Shandong Province

Cluster	County amount	Cases	Expected	Annual case/100000	RR	LLR	P value
***Total without adjusted for age (1970-1974)***							
Most likely cluster*	77	13103	10019	9.6	1.81	907.68	<0.001
Secondary cluster	7	1361	1192	8.4	1.15	12.10	<0.001
***Total adjusted for age(1970-1974)***							
Most likely cluster	77	10631	7898	7.8	1.96	906.73	<0.001
***Total without adjusted for age (2011-2013)***							
Most likely cluster	72	82388	71879	64.5	1.30	1374.05	<0.001
Secondary cluster 1	1	1928	1127	96.2	1.72	235.96	<0.001
Secondary cluster 2	2	4868	4060	67.5	1.21	77.58	<0.001
Secondary cluster 3	1	1267	894	79.8	1.42	69.39	<0.001
Secondary cluster 4	1	2029	1633	69.9	1.25	45.05	<0.001
Secondary cluster 5	1	2097	1785	66.1	1.18	25.95	<0.001
Secondary cluster 6	1	1518	1342	63.6	1.13	11.18	<0.01
***Total adjusted for age(2011-2013)***							
Most likely cluster	58	26161	23890	23.0	1.17	177.24	<0.001
Secondary cluster 1	5	3792	3102	25.6	1.24	75.74	<0.001
Secondary cluster 2	1	1436	1059	28.4	1.36	61.40	<0.001
Secondary cluster 3	1	524	333	33.0	1.58	46.89	<0.001
Secondary cluster 4	1	824	639	27.0	1.29	24.69	<0.001

Note *: “Most likely cluster” was defined when the maximum log likelihood ratio (LLR) with statistical significance was detected by Monte Carlo simulation in spatial analysis; the other LLR values with statistical significance were identified as “Secondary cluster”. The relative risk (RR) was the ratio of the mortality inside a cluster area to the mortality outside a cluster area.

The results of spatial scan statistical analysis showed that there were seven significant spatial clustering areas of lung cancer mortality rate in the entire province during 2011 to 2013 (Table 1 and Figure 5c). The most likely cluster was located in the northeastern region of Shandong Province, including 72 counties with a relative risk (RR) of 1.30 compared to the rest of Shandong Province. The secondary clusters were all located in southwestern region of Shandong, including 7 counties in six secondary clusters. The first secondary cluster included Dingtao County with an RR of 1.72. The second secondary cluster included Tengzhou County and Weishan County, with an RR of 1.21. The other secondary clusters each included one county, with RRs of 1.42, 1.25, 1.18 and 1.13 respectively. After age adjustment, six clusters were detected in the entire province and the spatial pattern had some differences compared with the spatial pattern before the age adjustment (Table 1 and Figure 5d). The most likely cluster was located in the northern region of Shandong Province, including 37 counties with a relative risk (RR) of 1.20 compared to the rest of Shandong Province. After age adjustment, 35 counties in the eastern region were no longer in the most likely cluster.

**Figure 6:**
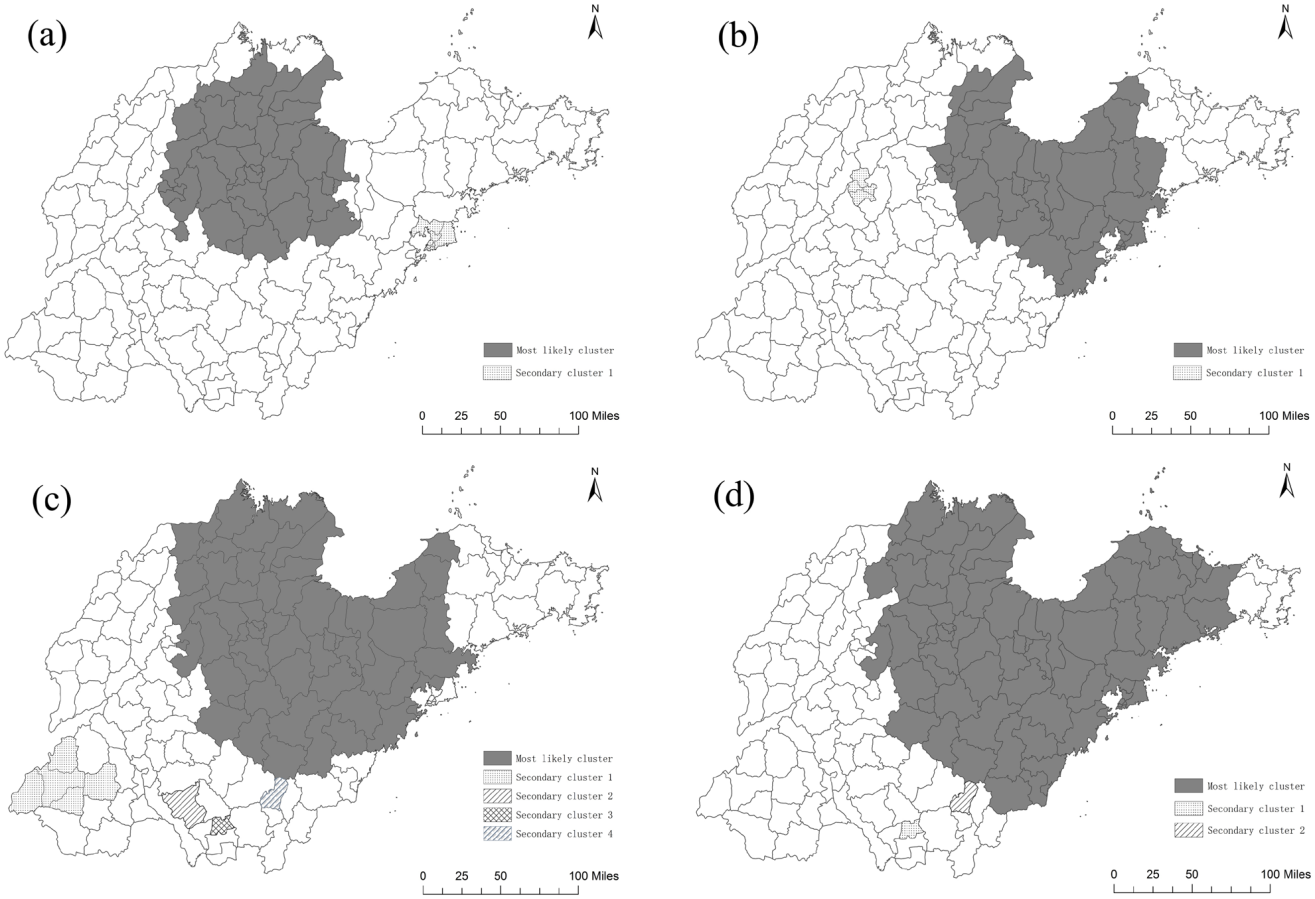
Lung cancer mortality clusters at the county level in Shandong for male and female during 1970-1974 and 2011-2013 using spatial scan statistical analysis **(a)** 1970-1974(ASMR for male), **(b)** 1970-1974(ASMR for female), **(c)** 2011-2013(ASMR for male), **(d)** 2011-2013(ASMR for female) ArcGis 10.2 (http://www.esri.com/software/arcgis/arcgisonline).

When we performed separate spatial scan statistical analyses for male and female, we found out that the most likely cluster was still located in the northern and eastern regions of Shandong Province. The counties in the most likely cluster of lung cancer mortality during 1970-1974 were still in the most likely clustering areas during 2011-2013 for both male and female (Figure 6). The number of counties in the most likely cluster reduced when separating the whole population by male and female in 1970-1974 (Figure 6a and 6b). But it changed a little in 2011-2013.

## DISCUSSION

Lung cancer has been the leading cause of cancer death in China [14] and had the fastest increase rate in death rate among all cancers in the past 40 years. The crude death rate had increased 6.88 times from 5.46 (1973-1975) to 43.03 per 100,000, which led to approximately 588,500 deaths in 2014 [15, 16]. In our study, the ARMR of lung cancer in Shandong had increased 6.81 times from 7.22 per 100,000 during 1970-1974 to 56.37 per 100,000 during 2011-2013. Although the increase rate of lung cancer mortality in Shandong was similar to the increase rate in China, the mortality rate of lung cancer in Shandong was significantly higher than the national average of China. Recent studies also showed an increasing trend in the lung cancer burden in China [17]. Chen et al. found a 1.63% per year increase in lung cancer incidence from 1988 to 2005, and the trend slowed down slightly after age adjustment [18]. Similar trend was also found by Fang et al. at an increase rate of 7.7% per year and a continued increase in the ensuing five years [19]. It was also reported that the sharp increase of lung cancer mortality was clearly out of proportion to the increase in population [20, 21]. In this study, we found out that the non-demographic factors contributed to 70.37% of the increase in lung cancer mortality rate in Shandong during 2011-2013, which was higher than that for Jiangsu Province(56.68%) shown by Zhou’s study [22]. This might be because the non-demographic factors (e.g., smoking, environmental air pollution and other risk factors) that caused the increase in lung cancer played a more significant role in Shandong Province.

Our previous study had explored the characteristics of regional distribution of cancer-related deaths in Shandong Province using principle components analysis [23]. We found out that the overall regional distribution of cancers in Shandong was dominated by several major cancers including lung cancer, esophagus cancer, stomach cancer and liver cancer. In this study, we find out that the spatial distribution of lung cancer mortality in Shandong Province is extremely uneven. During 1970-1974, the lung cancer mortality was higher in northern areas than that in southern areas of Shandong Province. Forty years later, most counties in the northern areas that used to be high in lung cancer mortality were still hotspots. This suggested that the risk factors for lung cancer in these areas are persistent and do not weaken or disappear.

The spatial statistical analysis developed by Kulldorff and Nagarwalla has been proved to be an effective method for discerning geographical patterns and detecting spatial clusters based on smaller units [24, 25]. In this study, we detected, for the first time, the spatial distribution and high-risk clusters of lung cancer mortality at the county level for a whole provincial population in China. We find out that the most likely cluster during 1970-1974 was located in the northern region of Shandong Province, around the Bohai Bay and including 77 counties with a relative risk (RR) of 1.81 compared to the rest of Shandong Province. After age adjustment, the most likely cluster did not change, still including the same 77 counties. During 2011-2013, the northern region of Shandong Province around the Bohai Bay was still the most likely cluster of lung cancer mortality, with or without age adjustment. As we know, Shengli Oil Field, which is the second largest oil field [26] in China (development began in 1961), is located in this region (mainly in Dongying). Around Shengli Oil Field, some other petrochemical enterprises were also established. Thus, the persistent clustering of lung cancer mortality in this region might be related to the relatively high concentration of petrochemical enterprises in the same region of Shandong Province. Some researchers [27, 28] had found out that residents near industrial areas are more likely to develop various diseases including lung cancer, which is consistent with the results of our research.

In this study, some secondary clusters were detected in the southwestern region of Shandong Province for the period of 2011-2013. These areas were low in lung cancer mortality previously and are mainly agricultural counties with a low level of industrialization. This is a new discovery, which will be studied further in the future.

Tobacco smoking and air pollution [29] have been identified as risk factors for lung cancer. About 80% of the lung cancer burden in men and 50% of the burden in women were attributed to these two risk factors. The incidence of lung cancer in China has increased during the past decades. Because of the high smoking rate for the past 20 years, lung cancer incidence in China may continue to increase for the next 10-20 years [30–32]. According to the Ministry of Health of China, most pollutant exposure occurs indoors, where people, especially women and children, spend the majority of their time [33]. Some common sources of indoor pollutants include tobacco smoke, second-hand smoke, cooking fumes, formaldehyde and benzene from decorative and building materials, and household products such as pesticides [34]. Previous studies had also identified household solid fuel (e.g., coal, which is used extensively in China for heating and cooking in homes, especially in rural areas) as a major source of household air pollution in Xuanwei [35], a rural county of China, attributing the high lung cancer mortality of this region to the heavy use of smoky coal [36]. According to our study, although the lung cancer mortality rate in male was much higher than that in female, the rising trend and attributable demographic and non-demographic factors were similar between males and females. This indicates, to some extent, that the increasing trend in lung cancer mortality might have little to do with the change of smoking rate, as the smoking rate in women was relatively low (all less than 3.1% since 2000 to 2013) in Shandong Province and the smoking rate had decreased slightly in Shandong Province from 2000 to 2013 [37–39]. Previous study also found out that lung cancer mortality rate in Chinese women was higher than the rate among women in some European countries despite a lower prevalence of smoking. This is thought to be caused by indoor air pollution from unventilated coal-fueled stoves and cooking fumes [40].

Some researchers also found out that the spatial distribution of air quality displayed a worsening trend from the coasts (eastern) regions to the inland (western) regions in Shandong Province [41, 42]. We found out that the clustering areas of lung cancer mortality were not located in regions with severe air pollution. In either 1970-1974 or 2011-2013, the clustering areas of lung cancer mortality in Shandong were not regions with severe air pollution. In addition, although the air condition changed remarkably in China over the past three decades [43], the most likely cluster of lung cancer mortality in Shandong Province had changed little over forty years. These findings suggested that the clustering of lung cancer mortality in Shandong Province might not correlate with outdoor air pollution, as air pollution was not an issue in China (including Shandong Province) over forty years ago. The clustering of lung cancer mortality in this area might be due to the relatively high concentration of petrochemical enterprises. On the other hand, indoor air pollution (including cooking fumes; formaldehyde, benzene and radon from decorative and building materials; household products such as pesticides, and so on) can also make lung cancer mortality increase. Further research such as case-control study is needed, and preventive measures can be carried out based on the research results.

In conclusion, this study explored the trend and determined the spatial distribution and clusters of lung cancer mortality in Shandong Province from 1970 to 2013. Lung cancer mortality increased remarkably regardless of whether age adjustment is performed or not. Demographic and non-demographic factors, in combination, caused such increase in lung cancer mortality. In addition, lung cancer mortality was not randomly distributed. A few significant spatial clusters of high lung cancer mortality were determined in Shandong Province at the county level. The most likely cluster was located in the northern region of Shandong Province both during 1970-1974 and during 2011-2013. This study revealed the mortality trend and provided evidence showing the presence of clusters of lung cancer mortality in Shandong, China. Such study may provide public health officials with the necessary information about the statistically significant clusters of lung cancer mortality, and thus enable them to carry out more effective and targeted strategies to reduce lung cancer incidence in different areas. More detailed individual-level investigation is needed for each of the identified clusters to evaluate potential determinants for the lung cancer mortality.

## MATERIALS AND METHODS

### Data collection

#### Shandong death registration system (SDRS)

Shandong Province, located in the east of mountain Tai-Hang and adjacent to the Bohai Sea and the Yellow Sea, has the second largest population among all provinces in China (Figure 3). Data on lung cancer caused deaths has been collected through official surveillance of the SDRS established in 2006. The SDRS, initially only covering the population of Disease Surveillance Points (DSPs), has been collecting data from the entire Shandong population since 2010 [44]. In this study, the case was defined as a death of residents in Shandong during 2011 to 2013 due to malignant neoplasm of lung cancer (C33, C34) according to International Classification of Diseases, 10^th^ reversion (ICD-10) [45] Supplementary Table 1. The death data was adjusted for underreporting based on underreporting surveys using the capture-mark-recapture method (CMRM) and official reports from the Household Registration System in China [46–48, 44]. In order to ensure data comparability, all quality control indicators such as the proportion of unknown cause of death, the under-reporting rate and the accuracy rate for ICD coding were all within the control range. CMRM is an epidemiologic method to estimate the size of targeted population with a specific characteristic [49]. It is hypothesized that M independent individuals in a randomly acquired sample from a targeted population with N individuals are marked and released to the original population. Another random sample with n independent individuals from the same targeted population is then acquired to identify the number of marked individuals (m). An unbiased formula is used to estimate the size of the targeted population based on the two independent samples. The population information used was from Shandong statistical yearbook, and age-specific sizes of age-specific population were acquired for 142 counties (cities/districts) in Shandong between 2011 and 2013.

#### National retrospective survey of mortality

In the mid-1970s, a nationwide retrospective survey of the causes of mortality was conducted in 29 provinces, including Shandong Province [50]. This survey covered all 122 cities and counties in Shandong Province for the period of 1970-1974. A national retrospective sampling survey of cancer mortality from 1990 to 1992 was also carried out. This survey employed a stratified sampling method, and covered approximately 10% of the population in China. 24 cities and counties from Shandong Province were enrolled as sampling areas [50, 51]. In 2006, a national retrospective stratified sampling survey of all causes of death for the period of 2004-2005 was conducted in 31 provinces/municipalities/autonomous regions, including Shandong Province [52]. 17 cities and counties from Shandong Province were selected as sampling areas. The sample representativeness of the second and third surveys in Shandong Province was good enough and this had been described in our previous publication [53].

### Statistical analysis

To reduce mortality variations in small populations and areas, an average reported mortality rate (ARMR) of lung cancer was calculated for each county (district) as a ratio of total deaths over corresponding population during the periods of 1970-1974, 1990-1992, 2004-2005 and 2011-2013. An age standardized mortality rate (ASMR) was calculated using the Chinese population in 1964. A difference decomposing method was applied to estimate the contribution of demographic and non-demographic factors for the change in ARMR in different eras [54]. The detailed formulae for the difference decomposing method can be found in previous publication [20]. The following formulae can help to better understand the method used.

The mortality rate in year b (Rb) -The mortality rate in year a (Ra)= Contribution value of demographic factor (Vd) + Contribution value of non-demographic factors (Vn). When the population structure in year a is taken as the standard population to calculate the standard rate in year b (Rbs), then Vn = Rbs - Ra and Vd = Rb - Rbs. The constituent ratio of demographic factor (Cd) =Vd/(Rb-Ra)×100%.

The ARMR and ASMR of lung cancer were illustrated using GIS-based maps at the county level to visualize the distribution patterns of lung cancer and high-risk (hotspot) areas in Shandong Province. The county-level point layer, including the latitude and longitude of the central point of each county, was created using GIS with a scale of 1:100,000 for the maps.

Purely spatial analyses using discrete Poisson model were performed to detect the spatial pattern of lung cancer in Shandong Province. The statistical significance of clustering was determined based on Monte Carlo hypothesis testing [55] by comparing the likelihood ratio test statistic from the observed data set with the test statistic from 999 random data sets generated under the null hypothesis of no clustering. The level of statistical significance was set as 0.05 in this study. Spatial Scan Statistics detect disease clusters by gradually scanning a window across space and comparing the numbers of observed and expected cases inside the window. In our study, we specified a maximum spatial cluster as one with 50% of the population at risk and conducted the scanning window in the shape of a circle. A most likely cluster was defined as a cluster with a maximum Log Likelihood Ratio (LLR) above the statistical significance. Secondary clusters that rejected the null hypothesis but do not overlap with the most likely cluster were also reported.

The calculation of lung cancer mortality (ARMR and ASMR) at the county level was conducted using Stata Version 12.0 (Stata Corp., College Station, TX, USA). County-level polygon maps of lung cancer clusters were drawn at a scale of 1:100,000 using ArcGis 10.2 (ESRI Inc., Redlands, CA, USA) [56]. Spatial Scan Statistics analysis was carried out to examine the presence of lung cancer clusters using SaTScan v9.1.1 developed by National Cancer Institute (NCI, Boston, MA, USA) [57].

### Study approval

The whole research protocol of this study has been approved by the Ethics Committee of Preventive Medicine in Shandong Center for Disease Control and Prevention in 2013, under permission No. 2013020. All methods including data collection and analysis method were performed in accordance with the relevant guidelines and regulations.

## SUPPLEMENTARY MATERIALS FIGURES AND TABLE



## Abbreviations

SDRS: Shandong Death Registration System
ICD-10: International Classification of Diseases, 10^th^ reversion
CMRM: capture-mark-recapture method
ARMR: average reported mortality rate
ASMR: age standardized mortality rate
RR: relative risk
LLR: log likelihood ratio
