# Supplementary material for: Lung cancer mortality clusters in Shandong Province, China: how do they change over 40 years?
Source: Oncotarget. 2017 Sep 21;8(51):88770–81. doi: 10.18632/oncotarget.21144 (PMC5687644; doi:10.18632/oncotarget.21144)
Supplement: Supplementary file 1 [file oncotarget-08-88770-s001.pdf]

Lung cancer mortality clusters in Shandong Province, China: how do they change over 40 years?

SUPPLEMENTARY MATERIALS

Smoking rate in Shandong Province

Since 2000, chronic diseases and their risk factors (including smoking status) have been regularly monitored for five times in Shandong Province by Shandong Center for Disease Control and Prevention. Smoking rates in Shandong Province in the year 2000, 2004, 2007, 2010 and 2013 were 31.6%, 27.6%, 26.7%, 23.8% and 24.0% respectively (Supplementary Figure 1). The smoking rate had decreased slightly in Shandong Province since 2000.

Supplementary Figure 2 shows the distribution of smoking rates in different investigated counties. It can be seen that the smoking rates in counties which are located in the most likely cluster of lung cancer mortality, such as Kenli County, Guangrao County and Lijin County, are not high. Therefore, the clustering of lung cancer mortality in this area may have nothing to do with smoking.

$PM_{2.5}$ concentration in Shandong Province

Supplementary Figure 3 shows the distribution of the average $PM_{2.5}$ concentrations in Shandong Province from 1998 to 2012. It can be found out that the $PM_{2.5}$ concentration reduced from the western interior to the eastern coast.

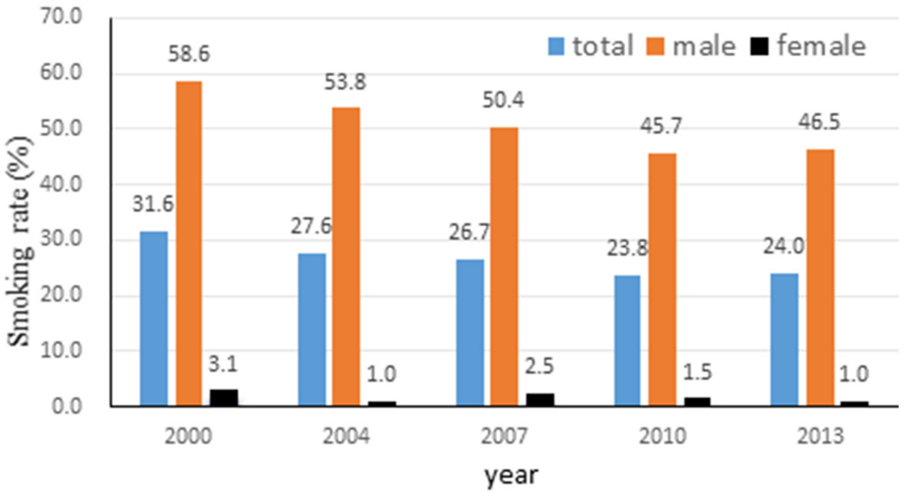

Supplementary Figure 1: Smoking rate in different years in Shandong Province.

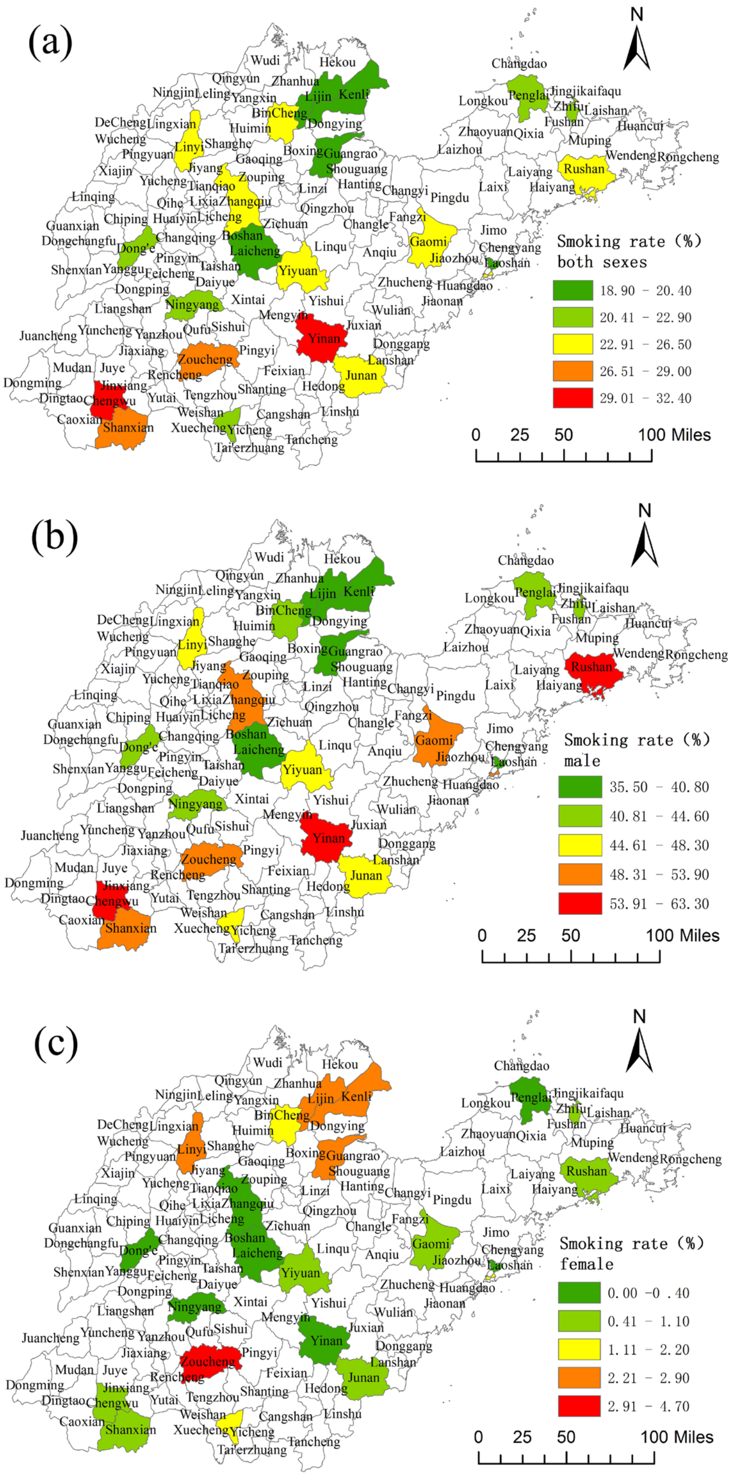

Supplementary Figure 2: Distribution of smoking rates of different sex in Shandong Province **(a)** both sexes, **(b)** male, **(c)** female.

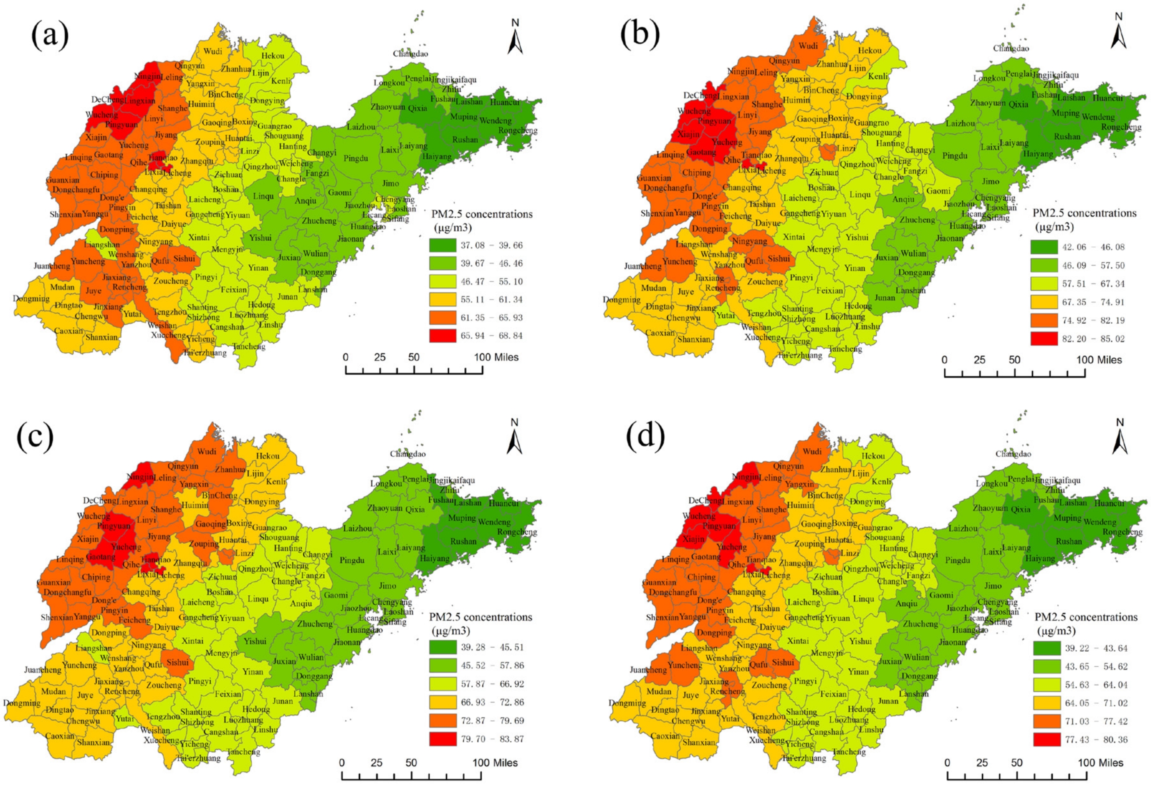

Supplementary Figure 3: Average PM2.5 concentrations in Shandong Province from 1998 to 2012 **(a)** 1998-2002, **(b)** 2003-2007, **(c)** 2008-2012, **(d)** 1998-2012.

Supplementary Table 1: Classification of malignant tumors and the ICD-10 code

Classification of malignant tumors	ICD-10 code
Lip, oral cavity and pharynx	C00-C14
Esophagus	C15
Stomach	C16
Colon and rectum	C18-C21
Liver	C22
Pancreas	C25
Throat	C32
Trachea, bronchus and lung cancers	C33-C34
Bone and articular cartilage	C40-C41
Malignant melanoma and other skin cancers	C43-C44
Breast	C50
Cervix	C53
Corpus uteri	C54-C55
Ovary	C56
Prostate	C61
Bladder	C67
Brain, CNS	C70-C71
Lymphoma and multiple myeloma	C81-C90, C96
Leukemia	C91-C95
Other cancers	C17, C23-C24, C26-C31, C37-C39, C45-C49, C51, C52, C57-C60, C62-C66, C68-C69, C72-C80, C97
